# Bridging the gap in bloodstream infection management: a survey among non-infectious disease physicians

**DOI:** 10.1093/jacamr/dlaf160

**Published:** 2025-09-11

**Authors:** Giovanni Mori, Emanuela Zandonà, Annalisa Campomori, Claudio Scarparo, Silvana Annamaria Urru, Gloria Stevanin, Giovanni Lorenzin, Marco Ripa, Alessandro Castelli, Alessia Prezzavento, Alberto Carli, Carla Lombardo, Massimiliano Lanzafame

**Affiliations:** Infectious Diseases Unit, Santa Chiara Hospital, Provincial Health Care Agency (APSS), Trento, Italy; Provincial Hospital Service Management, Provincial Health Care Agency (APSS), Trento, Italy; Hospital Pharmacy Unit, Santa Chiara Hospital, Provincial Health Care Agency (APSS), Trento, Italy; Microbiology Unit, Santa Chiara Hospital, Provincial Health Care Agency (APSS), Trento, Italy; Hospital Pharmacy Unit, Santa Chiara Hospital, Provincial Health Care Agency (APSS), Trento, Italy; Provincial Hospital Service Management, Provincial Health Care Agency (APSS), Trento, Italy; Microbiology Unit, Santa Chiara Hospital, Provincial Health Care Agency (APSS), Trento, Italy; Infectious Diseases Unit, Vita-Salute San Raffaele University, Milano, Italy; Infectious Diseases Unit, Santa Chiara Hospital, Provincial Health Care Agency (APSS), Trento, Italy; Infectious Diseases Unit, Santa Chiara Hospital, Provincial Health Care Agency (APSS), Trento, Italy; Medical Direction, Santa Chiara Hospital, Provincial Health Care Agency (APSS), Trento, Italy; Dermatology Unit, Santa Chiara Hospital, APSS, Trento, Italy; Infectious Diseases Unit, Santa Chiara Hospital, Provincial Health Care Agency (APSS), Trento, Italy; Center for Medical Sciences (CISMed), University of Trento, Trento, Italy

## Abstract

**Objective:**

Bloodstream infections (BSIs) significantly impact morbidity and mortality. Despite emerging evidence supporting optimal management, substantial variability persists among non-infectious disease (ID) physicians. This study assessed non-ID physicians’ knowledge and attitudes in BSI management, identifying critical gaps to inform antimicrobial stewardship (AMS) interventions.

**Methods:**

In December 2024, we conducted an online questionnaire among non-ID physicians at the Provincial Health Care Agency, Trento, Italy. An 18-item questionnaire, developed by a multidisciplinary group, evaluated key domains of BSI management, including diagnostic strategies, antibiotic selection, treatment duration, follow-up management, and ID consultation practices. Descriptive statistics were used to analyse response patterns.

**Results:**

Of 128 respondents, 99% expressed willingness to follow internal BSI guidelines, and 94% supported multidisciplinary feedback. Overall, 50.8% correctly identified the optimal 14-day antibiotic duration for uncomplicated *Staphylococcus aureus* bacteraemia (SAB), and 67.2% selected appropriate treatment for MSSA infections. The prevalence of complicated SAB was underestimated by 51.6% of participants. Follow-up blood cultures and echocardiography were variably recommended (40.6% and 71.9%, respectively, for SAB). 50.8% correctly indicated a 7-day therapy for uncomplicated Gram-negative BSIs, and 49.2% appropriately chose first-line treatments for susceptible *Enterobacterales*. Familiarity with antibiotic de-escalation (86.7%) and IV-to-oral therapy (94.5%) was high, but appropriate application knowledge was inconsistent. Penicillin safety in reported low-risk allergies was recognized by 63.3%, and carbapenems as alternatives by 46.1%.

**Conclusions:**

These findings highlights substantial knowledge gaps among non-ID physicians regarding bacterial BSI management. These findings support targeted AMS interventions under the Bacteraemia Evidence-based Active Treatment (BEAT) initiative to improve clinical outcomes.

## Introduction

Bacterial bloodstream infections (BSIs) significantly impact patient outcomes, causing approximately 2 million episodes and over 240 000 deaths annually in high-income countries.^1^  *Staphylococcus aureus* bacteraemia (SAB), particularly from MRSA, carries a particularly high mortality rate.^2^ Beyond acute mortality, BSIs can lead to long-term morbidity and functional decline.^3^ Optimal management includes timely pathogen identification, appropriate antimicrobial therapy, diagnostic evaluations for complications, and eventual source control.^4,5^ Although formal guidelines are lacking, except for MRSA,^6^ recent evidence supports best practices including antibiotic selection, treatment duration, follow-up blood cultures (FUBCs), and infectious disease (ID) consultations.^7^ However, adherence to these evidence-based practices varies even among ID specialists^8^ and is likely lower among non-ID physicians.

To assess attitudes and knowledge gaps, and to inform antimicrobial stewardship (AMS) interventions for BSIs, we conducted a survey among non-ID physicians. These findings prompted the Bacteraemia Evidence-based Active Treatment (BEAT) initiative, currently being implemented to harmonize BSI management across the institution in line with current evidence. The programme follows a bundle-of-care approach, incorporating concise internal guidelines and active multidisciplinary surveillance. Key qualitative and quantitative indicators have been selected to assess their impact over time.

## Methods

### Study design

This was an anonymous cross-sectional online survey conducted among non-ID physicians at the Provincial Health Care Agency (APSS) of Trento. Data collection occurred over a 4-week period from 24 December 2024 to 1 February 2025.

### Study population and sample selection

The target population included approximately 1400 non-ID physicians employed at APSS. Invitations were sent via email by the APSS director and department heads. Participation was voluntary, with no incentives. No structured education on BSI management had been provided prior to the questionnaire.

### Survey instrument

An 18-item questionnaire evaluated demographics, attitudes towards antimicrobial resistance and clinical guidelines, and clinical management of BSIs (*Streptococcus* spp., *S. aureus*, *Enterococcus* spp. and Gram-negative bacteria). It was developed by an AMS team (three ID physicians, one microbiologist, two pharmacists and two public health physicians). While not externally validated, the survey underwent internal validation by five independent ID specialists and was piloted for clarity and relevance.

### Statistical analysis

Descriptive statistics were performed using SAS software (version 9.4). Categorical data were summarized as absolute numbers and percentages. Univariate analyses found no significant associations between respondent demographics and survey responses (all *P* > 0.05). As all items were mandatory, only complete responses were analysed, with no missing data handling required.

## Results

A total of 128 physicians completed the questionnaire (mean age 44.5 ± 9.6 years; 57.8% from medical departments). Physicians indicated high priority for the availability of antimicrobial guidelines (median 10/10, rated top priority by 68%) and addressing antimicrobial resistance (median 10/10, rated top by 63.3%). Almost all (99%) would follow internal guidelines for BSI management, and 94% supported multidisciplinary feedback. Knowledge of optimal treatment varied: 50.8% correctly indicated a 14-day antibiotic course for uncomplicated SAB, and 51.6% underestimated the prevalence of complicated SAB. Only 67.2% correctly recognised oxacillin or cefazolin as the standard of care (SOC) for MSSA bacteraemia. Approximately 50% identified optimal treatment duration for uncomplicated Gram-negative BSI (GN-BSIs) and first-line antibiotics for susceptible *Enterobacterales*. Echocardiography was most frequently indicated for SAB (71.9%), followed by streptococci (64.8%) and *E. faecalis* (36.7%). Routine FUBCs were recommended for all BSIs by 44.5%. ID consultation was deemed essential for all BSIs by 54.7%. Most respondents were familiar with antibiotic de-escalation (86.7%), recognized its multiple benefits (90.6%) and agreed with IV-PO switch therapy in selected cases (94.5%). In cases of reported penicillin allergy, only 63.3% acknowledged that penicillins could often be safely used.

**Figure 1.**
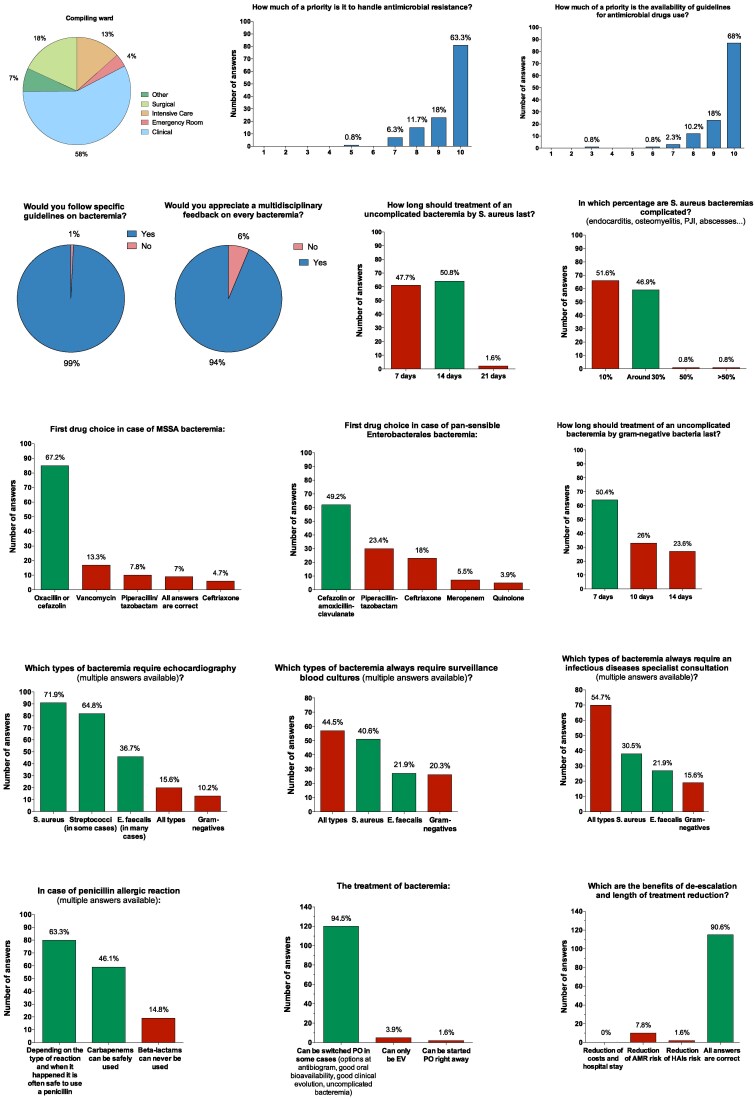
Survey responses on bloodstream infection management among non-infectious disease physicians.

Detailed responses are presented in Figure 1.

## Discussion

The analysis revealed key gaps in BSI management among non-ID physicians, particularly in treatment duration, first-line antibiotic choice and follow-up assessments (echocardiography, FUBCs, ID consultation).

Only 50.8% respondents correctly identified 14 days as the recommended duration for uncomplicated SAB, while 47.7% indicated 7 days. Such misperception risks compromising treatment efficacy and worsening outcomes in an already high-risk infection.^6^

The SNAP and CloCeBa trials support cefazolin as first-line therapy for MSSA. Preliminary, unpublished results from the SNAP (SYC Tong, JS Davis) and CloCeBa (X Lescure) trials were presented at the ESCMID Global 2025 session ‘The Trial Run: Recent Trials on *Staphylococcus aureus* Bacteraemia Management’ (Vienna, 13 April 2025). Although these results were not yet available at the time of the survey, only 67.2% of participants correctly indicated oxacillin or cefazolin as the SOC for MSSA. Vancomycin was inappropriately selected by 13.3%, despite its known inferiority to beta-lactams.^9^ Ceftriaxone (4.7%) and piperacillin-tazobactam (7.8%) were also chosen, despite guideline warnings^10^ and evidence of worse outcomes.^11^

Over half of respondents (51.6%) underestimated the prevalence of complicated SAB, assuming it to be 10%, despite evidence showing higher rates (5.7–75.3%) influenced by patient characteristics and diagnostic intensity.^12^ This may indicate a limited inclination to investigate for metastatic foci, with implications for recurrence and excess mortality.

Echocardiography was recommended by 71.9% of participants for SAB, 64.8% for streptococcal BSI, and 36.7% for *E. faecalis* BSI. The role of echocardiography is well established for SAB and streptococcal BSI (excluding *S. pyogenes* and *S. pneumoniae*),^13^ while recent studies showing a high IE prevalence support wider use in *E. faecalis* BSI,^14^ despite existing scores like NOVA and DENOVA.

FUBCs were recommended by 44.5% of respondents for all BSIs; 40.6% for SAB, 21.9% for *E. faecalis* BSI, and 20.3% for GN-BSIs. FUBCs are the SOC in SAB,^6^ where they help assess complication risk and guide treatment duration.^15^ Their use in *E. faecalis* BSI is supported by the high IE prevalence^14^ and may be warranted as part of care bundles.^16^ Their role in GN-BSIs remains debated: while meta-analyses of observational studies suggest a mortality benefit,^17–19^ the largest retrospective study found no such advantage and highlighted potential harms (including excess antibiotic use, longer hospitalization, and higher costs).^20^ Pending randomized controlled trials (RCTs), FUBCs should be reserved for patients with poor clinical response. In the future, validated risk scores may help identify GN-BSI patients who might benefit most.

ID consultation was deemed necessary for all BSIs by 54.7% of participants; specifically, 30.5% for SAB, 21.9% for *E. faecalis* BSI and 15.6% for GN-BSIs. For SAB, ID consultation is the SOC and often integrated into care bundles, with consistent evidence of reduced mortality, lower relapse risk and improved clinical management.^21^ Emerging data support its role also in enterococcal BSI and GN-BSIs.^16,22,23^ In enterococcal BSI, a large propensity score-matched study showed reduced mortality and improved care.^16^ In GN-BSIs, a systematic review demonstrated significant mortality benefits, particularly when consultation occurred early and in high-risk patients.^22^ Benefits likely stem from optimized antimicrobial use, improved diagnostic workup and more effective source control.

Only 50.8% of respondents correctly identified a 7-day course as appropriate for uncomplicated GN-BSIs, despite robust evidence from four RCTs^24–27^ and a target trial emulation^28^ supporting its non-inferiority to 14 days. A *post hoc* analysis of the most recent and largest trial (BALANCE) confirmed non-inferiority in high-risk subgroups and suggested a favourable trend in low-risk patients, supporting future trials on even shorter durations.^29^ The optimal duration for uncomplicated enterococcal BSI remains uncertain, with results from the ongoing INTENSE trial awaited.^30^

Only 49.2% of physicians correctly recognised amoxicillin–clavulanate or cefazolin (our local first-line agents) as the preferred first-line treatment for BSIs due to fully susceptible Enterobacterales, raising concerns about the ability of non-ID physicians to apply targeted therapy and antibiotic de-escalation effectively. This contrasts with the high reported familiarity with antibiotic de-escalation (86.7%) and its perceived benefits (90.6%). While evidence supporting antibiotic de-escalation in limiting antimicrobial resistance was historically limited, a recent large cohort study demonstrated its effectiveness, underscoring the importance of its implementation, particularly when microbiological data are available.^31^

Most respondents (94.5%) acknowledged the feasibility of switching to oral therapy for BSIs, aligning with growing evidence supporting early IV-PO transition. A recent meta-analysis of six RCTs and ten adjusted cohort studies (7102 patients) provided high-certainty evidence that early PO switch is non-inferior to continued IV therapy for treatment failure, and moderate-certainty evidence that it reduces hospital stay in uncomplicated BSIs.^32^ The SABATO trial further supported the PO switch in low-risk, uncomplicated SAB.^33^ Whether the IV-PO switch is safe and effective in complicated BSI remains to be proven.

While 63.3% of participants recognized that penicillins are often safe in patients with low-risk allergy, only 46.1% identified carbapenems as suitable alternatives. These findings contrast with current American and European guidelines,^34,35^ which recommend using structurally dissimilar beta-lactams or performing a monitored drug challenge without prior skin testing in adults with mild, non-anaphylactic reactions. This approach is further supported by the PALACE trial, which demonstrated the non-inferiority of a direct oral challenge in low-risk patients.^36^

To our knowledge, this survey is the first of its kind to examine key diagnostic and therapeutic attitudes towards BSIs among non-ID physicians. This study has several limitations. It lacked formal external validation and, as with most voluntary anonymous surveys, was inherently susceptible to selection, social desirability, and technology-access biases. Relevant demographic data were not collected, precluding subgroup analysis. Additionally, due to anonymity, convenience sampling, and the absence of a centralized recipient list, neither response rate calculation nor non-response analysis was feasible. Nonetheless, the questionnaire covered approximately 9% of APSS-employed physicians and included multiple departments, reasonably reflecting the institutional physician population.

## Conclusions

The study identified critical knowledge gaps among non-ID physicians in the management of BSIs, despite growing scientific evidence supporting best practices. Notably, incorrect treatment durations and suboptimal selection of first-line antibiotics raise concerns about both under- and overtreatment, including unnecessary use of broad-spectrum agents. Inconsistent application of follow-up diagnostics may result in missed complications and inefficient use of resources. While awareness of AMS principles appeared high, the findings suggest limitations in their consistent integration into clinical practice. These findings highlight the need for structured educational and organizational strategies to harmonize BSI management across specialties. The ongoing BEAT initiative is designed to address these challenges.

## Supplementary Material

dlaf160_Supplementary_Data

## Data Availability

The full survey is provided in the supplementary materials. Additional information is available upon reasonable request.

## Supplementary data

BEAT Survey is available as Supplementary data at *JAC-AMR* Online.
