# Supplementary material for: Bridging the gap in bloodstream infection management: a survey among non-infectious disease physicians
Source: JAC Antimicrob Resist. 2025 Sep 11;7(5):dlaf160. doi: 10.1093/jacamr/dlaf160 (PMC12455200; doi:10.1093/jacamr/dlaf160)
Supplement: dlaf160_Supplementary_Data [file dlaf160_supplementary_data.docx]

**BEAT (Bacteremia Evidence-based Active Treatment) initiative survey**

Anonymous survey to inform the design of an Antimicrobial Stewardship intervention aimed at simplifying and standardizing the hospital management of bacteremias.

All questions are mandatory. Estimated completion time: 10 minutes.

1. Age (in years): ….

2. Compiling ward: ☐ Medical ☐ Surgical ☐ Intensive Care ☐ Emergency Department ☐ Diagnostic and Support Services

3. How much of a priority is it to handle antimicrobial resistance? (Rate 1 - 10)

4. How much of a priority is the availability of guidelines for antimicrobial drugs use? (Rate 1 - 10)

5. Would you follow specific guidelines on bacteremia? ☐ Yes ☐ No

6. Would you appreciate multidisciplinary (ID specialist + microbiologist + pharmacist) feedback on every bacteremia? ☐ Yes ☐ No

7. How long should treatment of an uncomplicated bacteremia by *S. aureus* last? ☐ 7 days ☐ 14 days ☐ 21 days

8. In which percentage are *S. aureus* bacteremias complicated (e.g., endocarditis, osteomyelitis, infected prostheses, endophthalmitis, and/or abscesses)? ☐ 10% ☐ ~30% ☐ 50% ☐ >50%

9. What is the first-line agent for methicillin-susceptible *S. aureus* (MSSA) bacteremia? ☐ Oxacillin or Cefazolin ☐ Vancomycin ☐ Ceftriaxone ☐ Piperacillin-tazobactam ☐ All answers are correct

10. How long should treatment of an uncomplicated bacteremia by gram-negative bacteria last? ☐ 7 days ☐ 14 days ☐ 10 days ☐ Other

11. What is the first-line agent for a pan-sensible *Enterobacterales* bacteremia (e.g., *K. pneumoniae* or *E. coli*)? ☐ Cefazolin or Amoxicillin-clavulanate ☐ Piperacillin-tazobactam ☐ Quinolones ☐ Meropenem ☐ Ceftriaxone

12. Which types of bacteremia always require surveillance blood cultures (multiple answers available)? ☐ *S. aureus* ☐ *E. faecalis* ☐ Gram-negatives ☐ All types

13. Which types of bacteremia require echocardiography (multiple answers available)? ☐ *S. aureus* ☐ *E. faecalis* (in many cases) ☐ Gram-negatives ☐ Streptococci (in some cases) ☐ All types

14. Which types of bacteremia always require an infectious diseases consultation (multiple answers available)? ☐ *S. aureus* ☐ Enterococci ☐ Gram-negatives ☐ All types

15. In case of penicillin allergic reaction (multiple answers available): ☐ Beta-lactams can never be used ☐ Carbapenems can be safely used ☐ Depending on the type and timing of the reaction, penicillins can often be safely administered

16. Do you know what 'de-escalation' means? ☐ Yes ☐ No

17. What are the benefits of de-escalation and minimizing antibiotic duration? ☐ Reduction of costs and hospital stay ☐ Reduction of healthcare-associated infections (e.g., *C. difficile*, candidemia, catheter infections) ☐ Reduction of antibiotic resistance ☐ All answers are correct

18. The treatment of bacteremia: ☐ Can be switched PO in some cases (options at antibiogram, good oral bioavailability, good clinical evolution, uncomplicated bacteremia) ☐ Can only be EV ☐ Can be started PO right away
